# Circular RNAs in Cardiac Regeneration: Cardiac Cell Proliferation, Differentiation, Survival, and Reprogramming

**DOI:** 10.3389/fphys.2020.580465

**Published:** 2020-09-29

**Authors:** Julia Mester-Tonczar, Ena Hašimbegović, Andreas Spannbauer, Denise Traxler, Nina Kastner, Katrin Zlabinger, Patrick Einzinger, Noemi Pavo, Georg Goliasch, Mariann Gyöngyösi

**Affiliations:** ^1^Division of Cardiology, Department of Internal Medicine II, Medical University of Vienna, Vienna, Austria; ^2^Research Unit of Information and Software Engineering, Institute of Information Systems Engineering, Vienna University of Technology, Vienna, Austria

**Keywords:** circular RNAs, cardiac regeneration, cardiac cell proliferation, cardiac reprogramming, ischemic heart failure, cardiovascular disease

## Abstract

Circular RNAs (circRNAs) are classified as long non-coding RNAs (lncRNAs) that are characterized by a covalent closed-loop structure. This closed-loop shape is the result of a backsplicing event in which the 3' and 5' splice sites are ligated. Through the lack of 3' poly(A) tails and 5' cap structures, circRNAs are more stable than linear RNAs because these adjustments make the circular loop less susceptible to exonucleases. The majority of identified circRNAs possess cell‐ and tissue-specific expression patterns. In addition, high-throughput RNA-sequencing combined with novel bioinformatics algorithms revealed that circRNA sequences are often conserved across different species suggesting a positive evolutionary pressure. Implicated as regulators of protein turnover, micro RNA (miRNA) sponges, or broad effectors in cell differentiation, proliferation, and senescence, research of circRNA has increased in recent years. Particularly in cardiovascular research, circRNA-related discoveries have opened the door for the development of potential diagnostic and therapeutic tools. Increasing evidence links deviating circRNA expression patterns to various cardiovascular diseases including ischemic heart failure. In this mini-review, we summarize the current state of knowledge on circRNAs in cardiac regeneration with a focus on cardiac cell proliferation, differentiation, cardiomyocyte survival, and cardiac reprogramming.

## Introduction

Approximately 98% of the human genome is comprised of non-coding RNA (ncRNA) transcripts (Bär et al., 2020). Nevertheless, protein-coding genes remain the most well-studied sequences in the mammalian genome (Esteller, 2011). However, it became apparent that ncRNAs are crucially involved in a wide array of physiological and pathophysiological processes (Brennecke et al., 2003; Xu et al., 2003; Abbaszadeh-Goudarzi et al., 2020; Hashemian et al., 2020; Yousefi et al., 2020). One recently re-discovered class of ncRNAs is circRNAs.

The first description of circRNA coding organisms stems from 1976, when Sanger et al. examined viroids, circular single-stranded RNA pathogens of higher plants (Sanger et al., 1976). In eukaryotes, circRNAs were first detected in immortalized cervical cancer HeLa cells in 1979 using electron microscopy (Hsu and Coca-Prados, 1979). Until the 1990s, circRNAs were widely considered to be little more than splicing by-products or splicing errors (Capel et al., 1993; Cocquerelle et al., 1993). Since 2012, advances in high-throughput RNA-sequencing technology have identified circRNAs to be widespread, abundant and conserved across different species (Salzman et al., 2012, 2013; Jeck et al., 2013; AbouHaidar et al., 2014). Several circRNAs have shown a high degree of orthology between species as different as mice, pigs, and humans (Jeck et al., 2013).

CircRNAs were previously assumed to be present in low intracellular concentrations compared to conventional splicing products. However, recent analyses detected circRNAs in several human cell types and even indicated that they constitute a significant portion of the total spliced genetic product, varying between different cell types (Salzman et al., 2012). For some genes, the circular variant was also found to be more highly transcribed than the linear version (Salzman et al., 2012; Lasda and Parker, 2016).

In this mini-review, we provide a short overview on circRNA biogenesis and their mechanism of action in general. Further, we will summarize the newest findings regarding their role in cardiovascular diseases (CVD), in cardiomyocyte proliferation, differentiation, survival, and cardiac reprogramming. In conclusion, we outline current approaches to give insights into circRNAs in diagnostic and therapeutic settings, while considering state of the art methods, their limitations, and future challenges.

## To Come Full Circle: Biogenesis of CircRNAs

Canonical splicing is the default mode of splicing for linear pre-mRNA transcripts. In canonical splicing, an upstream (5') splice donor site is joined with a downstream (3') splice acceptor site (Ng et al., 2004). CircRNAs, however, are formed through non-canoncial splicing, where a downstream donor site is ligated with an upstream splice acceptor site (Braun et al., 1996). This type of splicing leads to the formation of a backsplice junction in circRNAs which therefore lack a 5' to 3' directionality. The resulting products are a covalently closed circRNA and an additional linear transcript with skipped exons that is subject to fast degradation (Chen and Yang, 2015).

CircRNAs have longer half-lives than linear RNAs because their closed-loop structure is resistant to exonucleases, which typically require a 3' or 5' end to initiate degradation (Jeck et al., 2013; Lasda and Parker, 2014; Enuka et al., 2016). The stability and the low degradation rates may imply that accumulation, rather than high production rates are accountable for the measured circRNA levels. The first sets of data regarding the expression patterns of circRNAs are becoming available. Although there is a general tendency for them to be expressed on a level similar to their linear counterpart, there are several exceptions, including the highly expressed circRNAs correlated to Titin (TTN) and ryanodine receptor 2 (RYR2; Tan et al., 2017). Further differences between circRNAs and linear RNAs are shown in Table 1.

**Table 1:** Main differences between circular RNAs (circRNAs) and linear RNAs.

	CircRNA	Linear RNA	References
Deep sequencing required	Yes	Yes	Salzman et al., 2012; Cooper et al., 2018; Das et al., 2019
Stability	Yes	No	Jeck et al., 2013; Lasda and Parker, 2014; Enuka et al., 2016
Exonuclease resistant^*^	Yes	No	Suzuki et al., 2006; Vincent and Deutscher, 2006; Salzman et al., 2012
Backsplice junction	Yes	No	Das et al., 2019
5' cap structure	No	Yes	Holdt et al., 2018
3' poly(A)-tails	No	Yes	Holdt et al., 2018
Length in basepairs (bp)	>200	21 to >200	Ding et al., 2018

^*^Some circRNAs are not exonuclease resistant (Szabo and Salzman, 2016; Legnini et al., 2017).

## Mechanism of Action

The length of circRNAs ranges from a few hundred to thousands of nucleotides (Chen, 2016) and their functions are versatile. Most studies on circRNAs focus on their ability to act as miRNA sponges, whereby they inhibit miRNA-mRNA binding (Hansen et al., 2013; Memczak et al., 2013). Further, study by Li et al. (2015) revealed that exon-intron circRNAs (EIcircRNAs) regulate gene expression in the nucleus by increasing the expression of their parental genes *via cis*-mediated mechanisms. In addition, intron-containing circRNAs (ciRNAs) have been shown to act as regulators of RNA polymerase II in cells by associating with the machinery responsible for the Polymerase II elongation and positively regulating its transcription (Zhang et al., 2013; Holdt et al., 2018). Even though they are generally classified as ncRNAs, studies have identifies a subset of circRNAs, which can be translated in a cap-independent manner (Legnini et al., 2017; Pamudurti et al., 2017). However, the exact mechanism of circRNA translation is yet to be fully elucidated. The majority of identified circRNAs are stably expressed, with cell‐ and tissue-specific expression patterns (Salzman et al., 2013; Ji et al., 2019). Because of the ability of cells to distinguish between endogenous and exogenous circRNAs based on the intronic sequence that initiates the circularization of the RNA during splicing (Chen et al., 2017), it follows that the recognition and degradation of invasive circRNAs is a regulated response of the immune system, with implications for autoimmune diseases (Chen et al., 2017; Zhong et al., 2019). Further pathologic conditions, in which their regulatory mechanisms have been thoroughly investigated include cancer (Shabaninejad et al., 2019; Naeli et al., 2020), neurologic disorders (Memczak et al., 2013), diabetes (Abbaszadeh-Goudarzi et al., 2020), and CVD (Wang et al., 2016; Werfel et al., 2016).

## CircRNAs in CVD

In recent years, the link between circRNAs and CVD has been studied intensively and indicates their involvement in the CVD pathogenesis (Viereck and Thum, 2017; Gurha, 2019; Huang et al., 2020). Since many circRNAs are highly conserved across species, animal models can be used to infer the role of circRNAs in human CVD (Werfel et al., 2016; Tan et al., 2017).

Two of the most well-known circRNAs with key functions in the heart, namely heart-related circRNA (HRCR) and CDR1as act as miRNA sponges (Huang et al., 2020). The conveyed effect depends on the “sponged” miRNA: while HRCR has an attenuating effect on hypertrophy by sponging miR-223 (Wang et al., 2016), CDR1as seems to amplify post-myocardial infarction (MI) ischemic damage in mice through sponging miR-7 (Geng et al., 2016). Interestingly, in a porcine model of MI increased expression of CDR1as was associated with reduced infarct size and increased left ventricular (LV) and right ventricular (RV) function (Mester-Tonczar et al., 2020). This might indicate interspecies differences in the role of CDR1as in CVD.

CircRNAs have been detected in peripheral fluids such as whole blood and plasma, where their increased chemical stability may be an advantage for their use as clinical biomarkers of diseases (Gurha, 2019). A study from 2019 established a circRNA-miRNA-mRNA network and identified circYOD-1 as a circulating biomarker for coronary artery disease (Miao et al., 2019). A study by Wu et al. (2019) identified three differentialy regulated circRNAs in pediatric patients with congenital heart disease: hsa_circRNA_004183, hsa_circRNA_079265 and hsa_circRNA_105039. Another study reported several circRNAs linked to hypertrophic cardiomyopathy (HCM) in humans. Three of the investigated circRNAs, DNAJC6, TMEM56, and MBOAT2 were found in serum and can help distinguish healthy from HCM patients. Furthermore, DNAJC6 and TMEM56 could serve as indicators for disease severity in patients with obstructive HCM (Sonnenschein et al., 2019). Identifying differentially regulated circRNAs through newly available sequencing technologies is the first step in planning further research with circRNAs.

## CircRNAs in Cardiac Regeneration: Cardiomyocyte Proliferation, Differentiation, and Survival

Despite essential advances in cardiac regenerative medicine, regeneration following myocardial ischemia still imposes many obstacles. Until recently, stem cell treatments, gene therapy, cell-based gene therapy, or the use of paracrine factors as regenerative cocktails of sorts represented the main focus of regenerative cardiology, yet the efficacy of such treatments failed to live up to expectations (Gyöngyösi et al., 2018). Regardless of the underlying etiology and the multitude of approaches for revascularizing or repairing ischemic tissue, reducing further damage outside the infarcted zone, and protecting the remaining healthy myocardium, the lack of clinically meaningful regenerative potential of mature cardiomyocytes and their replacement with non-functional scar results in an irreversible loss of functional tissue. Thus, new research directions focused on circRNAs in cardiac cell proliferation, differentiation, and cardiac survival should be examined.

The following part of the review focuses on current studies involving circRNAs within the context of the growing field of research on cardiac reprogramming. The natural starting point for any such investigations is the exploration of circRNA regulation in the growing and developing heart. Further investigations have focused on the differential regulation of circRNA transcripts in mesenchymal stem cells (MSCs) and induced pluripotent stem cells (iPSCs) during their experimentally induced differentiation into cardiomyocytes. Lastly, we discuss the literature on circRNA expression following MI.

### CircRNA Expression Patterns During Cardiac Development

In 2017, Li et al. examined the stage specific expression of lncRNAs and circRNAs in the undifferentiated mesoderm, cardiac progenitor, and definitive cardiomyocyte stages of cardiomyocyte development. The analysis uncovered important circRNA regulators, including circ-TTN, and a co-expression pattern of the circRNAs with genes such as MYL4, whose mutation has been linked to cardiac structural and electrical abnormalities (Li et al., 2017).

A 2017 study by Xu et al. measured the circRNA expression patterns in six human tissues, including the heart, and compared them to the expression patterns of the corresponding fetal tissue. As a general rule, they found circRNAs to be more highly expressed in the fetal stage of development. CircSLC8A1 was found to have a heart-specific upregulation pattern and might be a viable target for further research (Xu et al., 2017).

### CircRNAs in MSCs

MSCs not only have the ability to differentiate into cardiomyocytes, but can also recruit resident cardiac stem cells and secrete a variety of factors that could be useful in terms of cardiac regeneration (Williams and Hare, 2011).

A total of 226 differentially regulated circRNAs were discovered regarding differentiation of umbilical cord-derived human MSCs (huMSCs) into cardiomyocytes, with the most highly differentially regulated circRNAs related to differentiation and proliferation pathways, including the Wnt pathway (Ruan et al., 2019).

Another circRNA involved in the transcriptional preservation of stem cell identity is circFOXP1, whose silencing reduces huMSC growth and proliferation. MiR-17-3p and miR-127-5p are among the miRNAs sponged by circFOXP1. MiR-17-3p and miR-127-3p have a role in epidermal growth factor receptor (EGFR) and canonical Wnt signaling, an axis that allows them to interact in growth and survival pathways, as well as influencing the amount of differentiated MSCs (Cherubini et al., 2019).

CircRNA CDR1as has also been found to be abundant in huMSCs. The increase of huMSC proliferation, secretion, and differentiation induced by 3,3'-diindolylmethane also increased the abundance of CDR1as, whereas the knockdown of CDR1as reduces their proliferation and differentiation capacity, which are essential for their regenerative potential (Yang et al., 2019).

### CircRNAs in iPSCs

Early attempts at inducing cardiac regeneration focused on the stimulation of stem cell and cardiac progenitor cell populations naturally residing in the heart (Cai and Molkentin, 2017; Tzahor and Poss, 2017). However, this approach is limited by the small number of these c-kit+ and Sca-1+ cells in the adult myocardium and their replicative and functional limitations in replacing cardiomyocytes and achieving significant physiological effects (van Berlo and Molkentin, 2014; Cai and Molkentin, 2017).

Other approaches focused on the application of autologous or allogeneic somatic or embryonic stem cells. However, immunogenicity, engraftment, differentiation, tumogenicity, and ethical concerns were common issues with these methods.

A new era in the field of regenerative research, particularly in cardiac regeneration, was ushered in by Takahashi (Takahashi and Yamanaka, 2006; Takahashi et al., 2007). His first study revealed that differentiated somatic mouse cells can be reprogramed through the use of transcription factors to revert back to a pluripotent state (Takahashi and Yamanaka, 2006). The second study managed to replicate the same results in humans, inducing pluripotency in human dermal fibroblasts (Takahashi et al., 2007). These iPSCs became the focus of a multitude of studies and remain a very active field of research to this day. In a human induced pluripotent stem cell derived cardiomyocyte model, researchers investigated the differential regulation of circRNAs in cardiac development and identified 384 circRNAs that are specific to the setting of developing cardiomyocytes. Furthermore, multiple circRNAs were identified, which are relatively abundant or depleted in relation to the respective linear transcript over the course of cardiomyocyte differentiation, exemplified by circCDYL and circ-SMARCA5 (Siede et al., 2017).

A breakthrough study by Ieda et al. (2010) demonstrated that transformation of fibroblasts into functioning cardiomyocytes was successful, leading to the concept that differentiated somatic cells can directly be programed into another cell type without transitioning through the pluripotent stage.

### CircRNAs Following MI: A Story of Angiogenesis, Pluripotency, Proliferation, and Altered Outcomes

It is often difficult to speculate whether a single circRNA can convey a meaningful regeneration following a MI, since many circRNAs act on multiple pathways.

In 2019, Huang et al. identified super-enhancer associated circRNAs that could be relevant in regenerative pathways and further examined the role of circRNA Nfix in the regenerative capacity of the myocardium following a MI. On the one hand, circNfix knockdown not only induced increased cardiomyocyte proliferation in a murine model, but also resulted in improved angiogenesis, reduced infarcted and fibrotic area and improved cardiac functional parameters following an infarction. An induced overexpression of circNfix on the other hand decreased the ability of cardiomyocytes to proliferate *in vivo*. All of these potentially highly therapeutically relevant effects were found to be conveyed through promoting Ybx degradation and sponging miR-214 (Huang et al., 2019).

CircCDYL was the focus of a 2020 study on cardiac regeneration conducted by Zhang et al. This research group investigated circCDYL in adult mouse cardiomyocytes and in murine myocardium, and showed that overexpression of circCDYL *in vitro* promotes cardiomyocyte proliferation, whereas downregulation of circCDYL inhibits their proliferation. *In vivo* overexpression of circCDYL led to increase in ejection fraction (EF) in mice, whereas downregulation of circCDYL lowered the EF (Zhang et al., 2020b).

CircRNAs are also involved in post-infarction fibrotic remodeling that results in heart failure following MI in the long run. Zhu et al. (2019) investigated the role of circNFIB in cardiac fibrosis in a mouse model of MI and reported that circNFIB inhibition promotes adult fibroblast proliferation. Additionally, the sponge activity of circNFIB on miR-433 was also identified.

CircRNAs are often the topic of multidisciplinary research. CircHIPK3 is an example of a circRNA involved in cellular growth that has simultaneously been implicated in the growth, proliferation, and metastasis of cancer (Zheng et al., 2016; Zeng et al., 2018). CircHIPK3 is also relatively abundant in the fetal and neonatal myocardium of mice (Si et al., 2020, p. 3). This motivated an investigation into its potential regeneration-inducing capacity in the myocardium *in vitro* and *in vivo*. CircHIPK3 is perhaps the perfect example to illustrate the sheer multitude of effects that can be exhibited by a single circRNA. Si et al. (2020) demonstrated that the overexpression adenoviral vector-associated circHIPK3 induces coronary artery endothelial proliferation, increases cardiomyocyte proliferation, promotes angiogenesis, and decreases fibrosis in the zone surrounding the infarction area in adult mice. Whereas the pro-angiogenic properties seem to be conveyed through a miRNA-133a sponging effect, the effects on the cardiomyocytes were found to be achieved through an increase in the stability of the Notch1 intracellular domain. Table 2 summarizes circRNAs involved in cardiomyocyte proliferation, differentiation, and cardiac reprogramming.

**Table 2:** CircRNAs and their role in cardiac cell proliferation, differentiation, and cardiac reprogramming.

CircRNA	Regulation	Mechanism	Consequence	Type of sample (number of samples)	References
HRCR	Overexpression	miR-223 sponge	Repression of cardiac hypertrophy and HF in mice	Mice hearts (*n* = 4–8/group) Adult human hearts (*n* = 7–8/group)	Wang et al., 2016
CDR1as	Increased expression	Currently unknown	Reduced infarct size and positive influence on LV and RV function	Pig hearts (*n* = 5/group)	Mester-Tonczar et al., 2020
CDR1as	Overexpression	miR-7 sponge	Increased infarct size in a mouse model	Mice hearts (*n* = 10/group)	Geng et al., 2016
CDR1as	Knockdown	Currently unknown	Depleted proliferation and differentitation capacity of huMSCs and induced cell apoptosis	huMSCs	Yang et al., 2019
circFOXP1	Silencing circFOXP1	miR-17-3p sponge and miR-127-5p sponge resulting in the modulation of non-canonical Wnt and EGFR pathways	Reduction of huMSC growth and proliferation	huMSCs	Cherubini et al., 2019
circNfix	Downregulation	Promotes Ybx1 ubiquitin-dependent degradation and miR-214 sponge	Cardiomyocyte proliferation and inhibition of cardiomyocyte apoptosis after MI in mice	Mice hearts (*n* = 2–10/group) Adult human hearts (*n* = 2)	Huang et al., 2019
circCDYL	Overexpression	Currently unknown	Improved heart function after AMI	Myocardial tissue of mice (*n* = 6) Hypoxic cardiomyocytes	Zhang et al., 2020b
circNFIB	Overexpression	miR-433 sponge	Attenuation of cardiac fibroblast proliferation	Mice hearts (*n* = 5–6/group) TGF-beta treated cardiac mice fibroblasts	Zhu et al., 2019
circHIPK3	Overexpression	Increased N1ICD acetylation and miR-133a sponge	Induction of coronary artery endothelial cell proliferation, promoted cardiomyocyte proliferation and angiogenesis, and decreased fibrosis after MI	Mouse model of MI (*n* = 6)	Si et al., 2020

huMSCs, human mesenchymal stem cells; HF, heart failure; LV function, left ventricular function; MI, myocardial infarction; N1ICD, Notch1 intracellular domain; RV function, right ventricular function. The number of samples varies in these studies, because the research groups used varying numbers of animals for each of their experiments.

## Areas of Application

As CircRNAs are involved in cell differentiation and proliferation as well as regulating transcription (Bose and Ain, 2018) and translation (Pamudurti et al., 2017), their potential applications are plentiful. CircRNAs are currently the focus of a number of biomarker studies (Ouyang et al., 2017; Zhao et al., 2017; Li et al., 2018), due to their chemical stability compared to other RNA molecules (Jeck et al., 2013). Wesselhoeft et al. (2018) demonstrated the use of circRNAs for robust and stable translation in eukaryotic cells. This innovation offers great possibilities for obtaining stable protein production for biotechnological use. Their development can be used as an alternative to mRNA translation as mRNAs display relatively short half-lives compared to circRNAs. Another area of application is the construction of synthetic miRNA sponges. Liu et al. (2018) demonstrated a successful designing of a synthetic sponge constructed out of a circularized product targeting miR-21. As a consequence, gastric carcinoma cell proliferation was inhibited. In the past 3 years, other studies also reported the engineering of cost-effective artificial miRNA sponges (Rossbach, 2019; Wang et al., 2019). These examples illustrate the potential of circRNA for developing biomarkers, tools for circRNAs translation, and therapeutic potential for regulating cell proliferation.

## Challenges in CircRNA Research

Since most circRNAs show low expression patterns (Zhang et al., 2020a), their detectability requires state of the art methods. For the detection of novel circRNAs deep-sequencing paired with bioinformatics algorithms specialized on detecting backsplice junctions is required. Several algorithms exist, each with its own advantages and disadvantages. Some algorithms like KNIFE, CIRCexplorer, and CIRI achieved the best sensitivity, whereas others like UROBORUS can detect low expression levels of circRNAs in rRNA depleted samples without RNase R digestion (Chen et al., 2020). No poly(A) enrichment should be performed before RNA-sequencing and a read length of at least 100 basepairs is recommended to accurately align the reads with the backsplice junction (Kristensen et al., 2019). Some laboratories propose performing an RNase R digestion for circRNAs, however, not all circRNAs are resistant to RNase R (Szabo and Salzman, 2016; Legnini et al., 2017). Instead of using RNase R in circRNA research, one could use divergent primers spanning the backsplice junction and confirm the circular transcript with Sanger sequencing. Similarly, RNA-sequencing data can be validated through Sanger sequencing as well.

## Conclusion

In this review, we summarized some of the recent findings on the topic of circRNAs and their role in cardiomyocyte proliferation, differentiation, survival, and cardiovascular regeneration. Given the fact that circRNAs were only recently re-discovered compared to other ncRNAs, the full potential of circRNAs has yet to be elaborated.

## Author Contributions

JM-T wrote the manuscript and put the tables together. MG, EH, and AS helped in literature search and assisted with writing the manuscript. DT, NK, KZ, PE, NP, and GG edited the manuscript and the tables and helped to structure and revise the manuscript. All authors contributed to the article and approved the submitted version.

### Conflict of Interest

The authors declare that the research was conducted in the absence of any commercial or financial relationships that could be construed as a potential conflict of interest.
